# Draft Genome Sequence of *Winogradskyella psychrotolerans* RS-3^T^, Isolated from the Marine Transect of Kongsfjorden, Ny-Ålesund, Svalbard, Arctic Ocean

**DOI:** 10.1128/genomeA.00630-13

**Published:** 2013-08-15

**Authors:** Anil Kumar Pinnaka, Sreenivas Ara, Aditya Singh, S. Shivaji

**Affiliations:** CSIR-Institute of Microbial Technology, Chandigarh, Indiaa; CSIR-Centre for Cellular and Molecular Biology, Habsiguda, Hyderabad, Andhra Pradesh, Indiab

## Abstract

The 4.3-Mb genome of *Winogradskyella psychrotolerans* strain RS-3^T^, isolated from a sediment sample of a marine transect of Kongsfjorden, Ny-Ålesund, Svalbard, Arctic Ocean, is reported.

## GENOME ANNOUNCEMENT

*Winogradskyella psychrotolerans* strain RS-3^T^ is a Gram-negative, nonmotile, rod-coccus shaped bacterium. It was isolated from a marine transect sample from Kongsfjorden, Ny-Ålesund, Svalbard, Arctic Ocean. *Winogradskyella pacifica* and *Winogradskyella thalassocola* were reported to be the most closely related species by the 16S rRNA gene sequence analysis, with sequence similarities to the type strains of these species of 98.5 and 97.7%, respectively (1).

The Roche 454 (FLX Titanium) pyrosequencing platform was used to sequence the genome of *W. psychrotolerans* strain RS-3^T^. The sequencing yielded 121,341,791 bases in 288,329 reads, which is 28× coverage of the genome. The GS *de novo* assembler (v2.8) was used for the assembly of the raw sequencing data, which yielded a genome of 4,342,100 bp in 219 contigs, with a G+C% mol of 37.70%. The genome annotation was performed using the Rapid Annotations using Subsystems Technology (RAST) server (2), tRNAs were predicted by tRNAscan-SE (v1.23) (3), and rRNA genes were predicted by RNAmmer (v1.2) (4).

The annotation has a total of 4,068 genes, including 44 RNA genes. The annotation has 54 genes providing resistance against different antibiotics and toxic compounds. There are 107 genes for membrane transport, including 5 ABC transporters and 5 cation transporters. The genome also has 67 genes for different stress responses, including 33 genes for oxidative stress, 14 heat shock response genes, and 5 cold shock response genes.

A functional comparison of the genome was performed using the SEED framework (4) which revealed the closest neighbors of *W. psychrotolerans* RS-3^T^ to be *Flavobacteriales bacterium* ALC-1, *Croceibacter atlanticus* HTCC2559, *Cellulophaga* sp. MED134, *Dokdonia donghaensis* MED134, and *Zunongwangia profunda* SM-A87, with similarity score values of 513, 391, 358, 344, and 343, respectively.

Whole-genome sequencing of this bacterium will help in understanding the metabolism and evolution of bacteria under extreme weather conditions, like the Arctic Ocean.

### Nucleotide sequence accession numbers.

The draft genome sequence of *W. psychrotolerans* RS-3^T^ has been deposited in DDBJ/EMBL/GenBank under the accession no. ATMR00000000. The version described in this paper is the first version, ATMR01000000.
